# Low-kilovolt x-ray intraoperative radiotherapy for pT3 locally advanced colon cancer: a single-institution retrospective analysis

**DOI:** 10.1186/s12957-020-01903-x

**Published:** 2020-06-17

**Authors:** Li Ma, Junhao Qiang, Heliang Yin, Lin Lin, Yan Jiao, Changying Ma, Xinwei Li, Li Dong, Jinglin Cui, Dongmei Wei, Ankur M. Sharma, David L. Schwartz, Weikuan Gu, Hong Chen

**Affiliations:** 1 Center of Integrative Research, The First Hospital of Qiqihar, 30 Gongyuan Road, Longsha District, Qiqihar, 161005 Heilongjiang People’s Republic of China; 2 Affiliated Qiqihar Hospital, Southern Medical University, 30 Gongyuan Road, Longsha District, Qiqihar, 161005 Heilongjiang People’s Republic of China; 3 Department of Orthopedic Surgery and BME-Campbell Clinic, University of Tennessee Health Science Center, 956 Court Avenue, Memphis, TN 38163 USA; 4 Department of Radiation Oncology, University of Tennessee Health Science Center, 920 Madison Avenue, Memphis, TN 38163 USA; 5 Research Service 151, VA Medical Center, 1030 Jefferson Avenue, Memphis, TN 38104 USA

**Keywords:** Low-kV x-ray, Intraoperative radiotherapy, Locally advanced colon cancer, pT3 colon cancer

## Abstract

**Background:**

Patients with locally advanced colon cancer (LACC) treated with surgery had a high risk of local recurrence. The outcomes can vary significantly among patients with pT3 disease. This study was undertaken to assess whether low-kilovolt (kV) x-ray intraoperative radiotherapy (IORT) can achieve promising results compared with electron beam IORT (IOERT) and whether specific subgroups of patients with pT3 colon cancer may benefit from low-kV x-ray IORT.

**Methods:**

We retrospectively reviewed 44 patients with pT3 LACC treated with low-kV x-ray IORT. Clinicopathologic characteristics were analyzed to identify patients that could potentially benefit from low-kV x-ray IORT. The Kaplan-Meier survival analysis was used to assess overall survival (OS) and progression-free survival (PFS). Correlation analysis was used to discover the association of multiple factors to the results of treatment represented by the values of OS and PFS.

**Results:**

The median follow-up of patients was 20.5 months (range, 6.1–38.8 months). At the time of analysis, 38 (86%) were alive and 6 (14%) had died of their disease. The 3-year Kaplan-Meier of PFS and OS for the entire cohort was 82.8% and 82.1%, respectively. At median follow-up, no in-field failure within the low-kV x-ray IORT field had occurred. Locoregional and distant failure had occurred in 2 (5%) patients each. The rate of perioperative 30-day mortality was 0%, and the morbidity rate was 11%. Five patients experienced 7 complications, including 4 early complications (30 days) and three late complications (> 30 days) leading early and late morbidity rates of 9% and 7%, respectively.

**Conclusion:**

Patients with LACC who had undergone an additional low-kV x-ray IORT can achieve encouraging locoregional control, PFS, OS, and distant control without an increase in short-term or long-term complications. Low-kV x-ray IORT can be considered as part of management in pT3 LACC.

## Background

Colon cancer was the fourth most common malignancy and the fifth most deadly cancer worldwide according to the GLOBOCAN estimation in 2018 [1]. Completeness of surgical resection was the most important prognostic factor in almost all the studies [2]. Most colon cancer patients were sufficiently treated surgically with or without adjuvant systemic therapy. Although 70 to 90% of all patients who had colorectal cancer undergo surgical resection with curative intent, the 5-year recurrence rate was 12% and 33% in stage II and III patients, respectively [3, 4]. Multivariable analysis indicated that disease stage II and III were independent predictors of locoregional recurrence (LR). The median survival after diagnosis of LR was only 9 months [5]. Consequently, the recurrence or metastasis leads to a clinical and therapeutic challenge associated with a poor prognosis. It is therefore worth exploring how local control could be improved beyond standard care of colon cancer.

At present, there is no established role for the routine use of intraoperative radiotherapy (IORT) as adjuvant therapy in primary colon cancer except for in pT4 disease. However, IORT allows for sterilization of microscopic disease in situ. Shifting healthy tissues out of the radiation field and selective shielding of surrounding structures during IORT, therefore the high, single radiation doses, can be delivered while minimizing the side effects in adjacent tissues [6]. Studies have indicated that modification of IORT for colorectal cancers may lead to an improvement of in-field and local control in selected patients [7–9]. Brady et al. have reported that IORT may be utilized as a tool to improve local control in patients with locally advanced primary or recurrent colorectal cancer [10]. However, there were limited previous studies of IORT for primary colon cancer, and most of the patients in these researches received either IOERT or high-dose-rate intraoperative brachytherapy [11, 12] with only a few studies describing outcomes for colorectal cancer patients using orthovoltage IORT [13–15]. At present, electronic brachytherapy is mainly recommended for breast cancer, endometrial, cervical cancer, or non-melanomatous skin cancers based on currently available data; however, electronic brachytherapy has emerged as a potential alternative for certain disease sites [16].

Currently recognized high-risk factors for recurrence of colon cancer after resection included poorly differentiated histology, lymphatic/vascular invasion, perineural invasion, or positive margins. To explore patients who would benefit from low-kV x-ray IORT in pT3 patients, we analyzed the data based on the clinicopathological characteristics of the patients. This study is the first time to investigate potential benefit from low-kV x-ray IORT among patients with pT3 LACC. We aim to evaluate whether low-kV x-ray IORT can benefit pT3 patients not being inferior to the electron IOERT. Furthermore, we report complications associated with low-kV x-ray IORT.

## Methods

The local institutional review board approved this study. We retrospectively analyzed clinical data of 44 primary colon cancer patients with T3N0-2 M0 diseases. They all received curative surgical resections and low-kV x-ray IORT at our hospital between August 2016 and February 2019. A tumor within 15 cm from the anal verge at the caudal margin defined as rectal cancer was excluded. We also excluded distant metastasis, recurrent colon cancer, and synchronous malignancy.

Standardized curative intent surgeries were applied in all patients. We restaged the final pathologic features according to the tumor node metastasis (TNM) staging system of the seventh edition of the American Joint Committee on Cancer during data review.

Lower energy photons were performed to the tumor bed, while dose-limiting structures were separated from the irradiation field. It was applied using a dedicated INTRABEAM® PRS 500 (Carl Zeiss Meditec AG, Germany). The operation of the INTRABEAM system was based on the use of an orthovoltage x-ray beam (photons with an energy of 50 kV). The diameters of spherical applicators ranged from 1.5 to 5.0 cm. They were used to enable accuracy and uniformities of dose distribution on the surface of the tumor bed. The dose adjustment is dependent on the proximity of surrounding risk structures (e.g., Peripheral nerve is dose-limiting for intraoperative radiotherapy, and patients receiving 15 Gy or more are at higher risk.) and the degree of adhesion of the tumor to the surrounding tissue during surgery. Higher doses (≥ 15 Gy) were delivered due to a close or positive margin. Our study was designed based on the experience of other institutes on the IORT dose administration [17–19].

The Kaplan-Meier survival analysis was used to assess overall survival (OS) and progression-free survival (PFS). Comparison was done using ANOVA analysis; *P* value less than 0.05 was considered as significant. Correlation analysis was used to discover the association of multiple factors to the results of treatment represented by the values of OS and PFS. For the *r* value equal or more than 0.7 or − 0.7, we treated it as significant correlation. For an *r* value between 0.35 and 0.69 or − 0.35 and − 0.69, we regard it as existence of a correlation. When *R* values fell between 0 and 0.35 or 0 and − 0.35, we regarded these data as no correlation. Statistical analyses were performed with the SPSS 26.0 statistical software (IBM SPSS Statistics 26).

## Results

Twenty-eight men (64%) and sixteen women (36%) were included in this study. Median age at the time of surgery and low-kV x-ray IORT was 64.5 years (range 39–83). One patient had small intestinal neuroendocrine carcinoma, three had mucinous adenocarcinoma, and all others had adenocarcinoma. Postoperative chemotherapy was administered to nineteen patients according to postoperative pathology. Except for two patients who used capecitabine for 3–8 cycles, the remaining 17 patients received regimen CAPEOX for 3–6 cycles. Additional information on patient and tumor characteristics is described in Table 1. The information on low-kV x-ray IORT is described in Table 2.

**Table 1:** Patient and tumor characteristics (*n* = 44)

Characteristic	Number (%)
Sex	
Male	28(64%)
Female	16(36%)
Age (years)	
Median	64.5
Range	39–83
Preoperative RT	0
Neoadjuvant chemotherapy	0
Postoperative RT	0
Adjuvant chemotherapy	19
Histology	
Adenocarcinoma	40 (91%)
Mucinous adenocarcinoma	3 (7%)
Small intestinal neuroendocrine carcinoma	1 (2%)
Pathologic T stage	
T3	44 (100%)
Pathologic N stage	
N0	27
N1	11
N2	6
Pathologic M stage	
M0	44
M1	0
Number lymph nodes examined	
Median	19
Range	7–33
Number lymph nodes positive	
Mean	2
Range	0–15
Follow-up time (months)	
Median	20.5
Range	6.1–38.8

**Table 2:** Mean outcome and range by low-kV x-ray IORT characteristics

Characteristic	Number (%)
Applicator size (cm)	
Mean	3.5
Range	2.5–4.5
Dose (Gy)	
Mean	15
Range	10–18
Time (min)	
Mean	15.7
Range	9.2–26.6

**Fig. 1:**
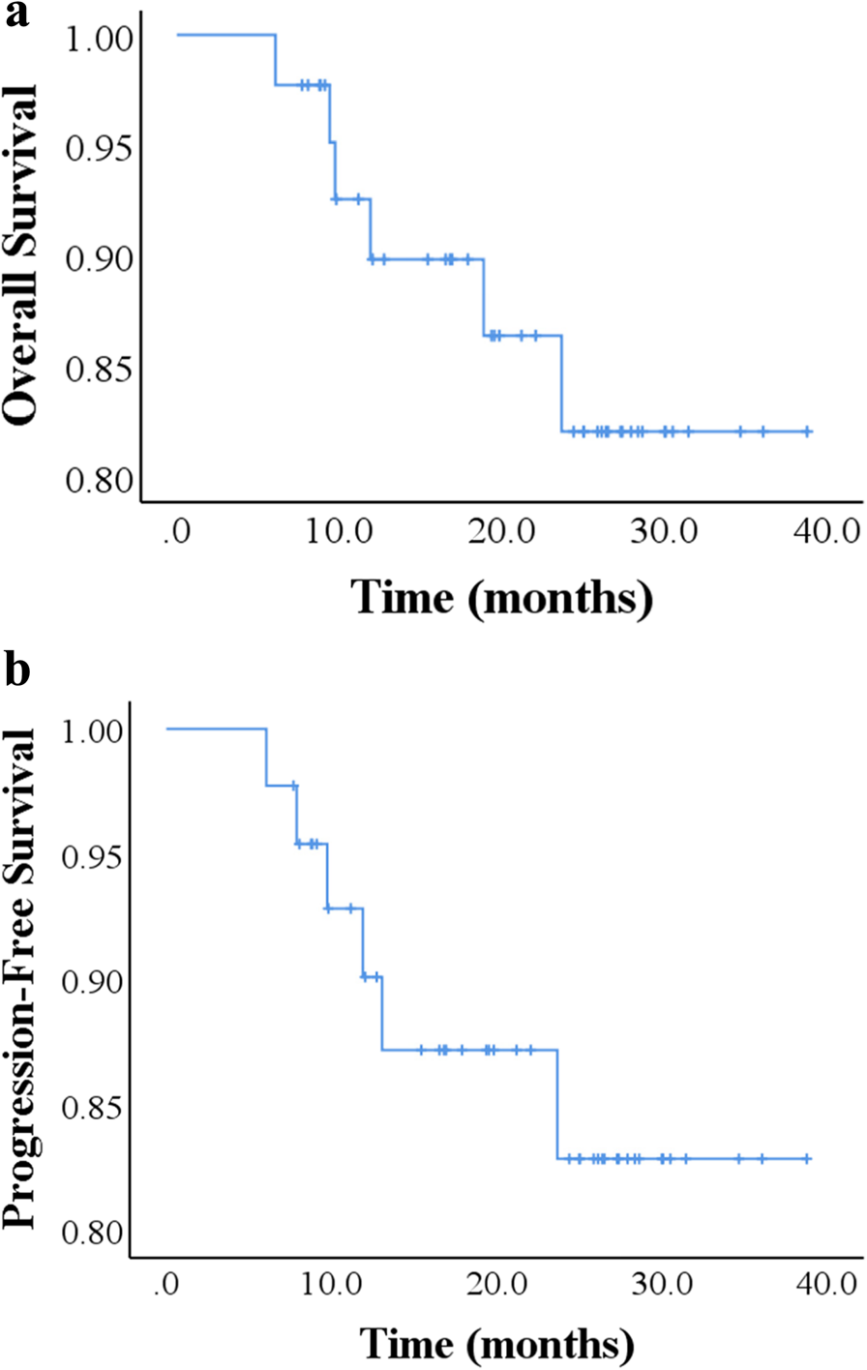
**a** Kaplan-Meier overall survival curve; **b** Kaplan-Meier progression-free survival curve

Median follow-up of patients was 20.5 months (range, 6.1–38.8 months). At the time of analysis, 38 (86%) of 44 patients were alive, and 6 (14%) patients were dead. The 3-year Kaplan-Meier of OS and PFS for the entire cohort was 82.1% and 82.8%, respectively (Fig. 1a, b). At median follow-up, no central failure within the low-kV x-ray IORT boost field had occurred, and locoregional and distant failure had occurred in 2 (5%) patients each.

**Fig. 2:**
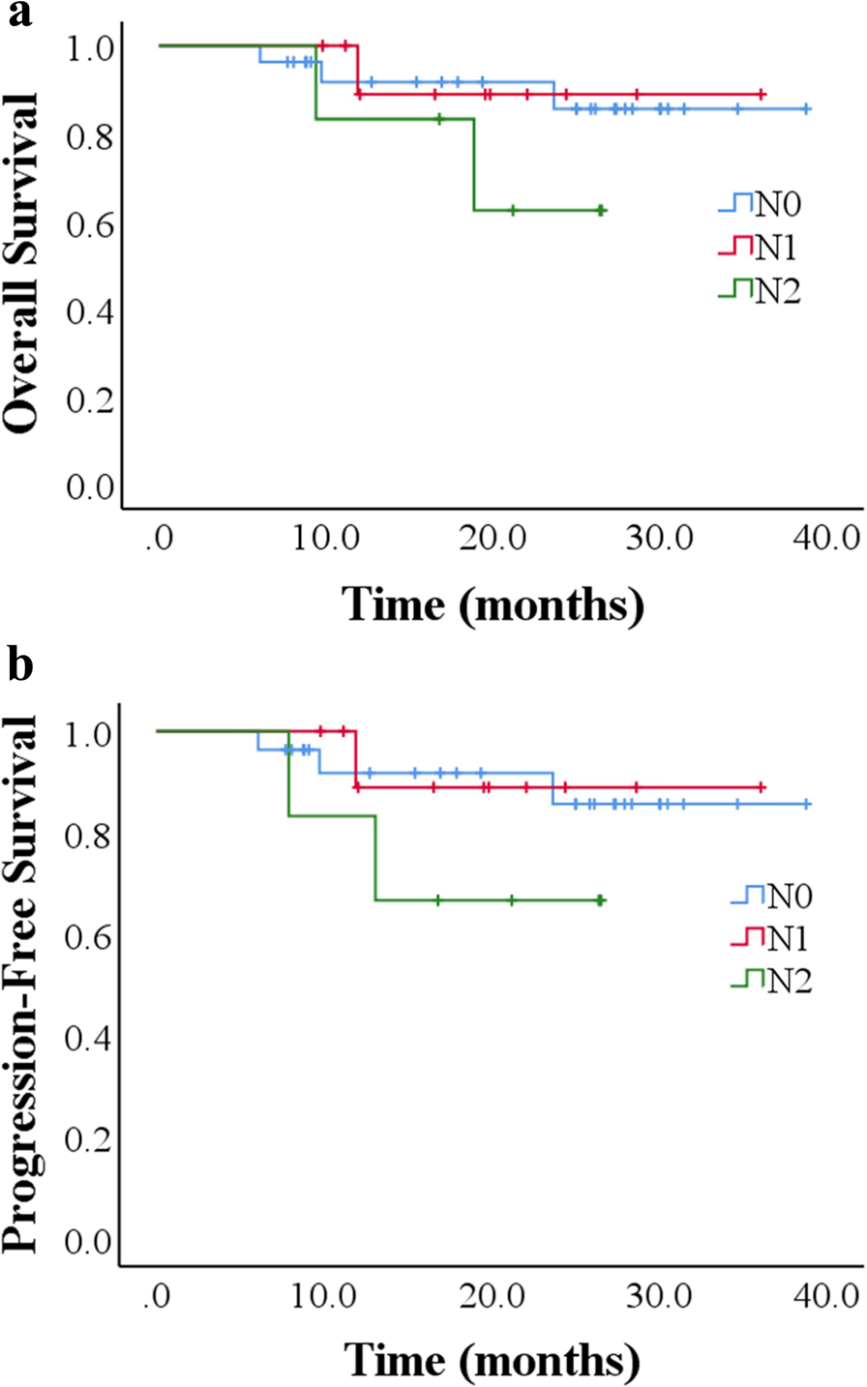
**a** Kaplan-Meier overall survival curve (*p* = 0.38); **b** Kaplan-Meier progression-free survival (*p* = 0.373). Patients were stratified by pathologic regional lymph node status

**Fig. 3:**
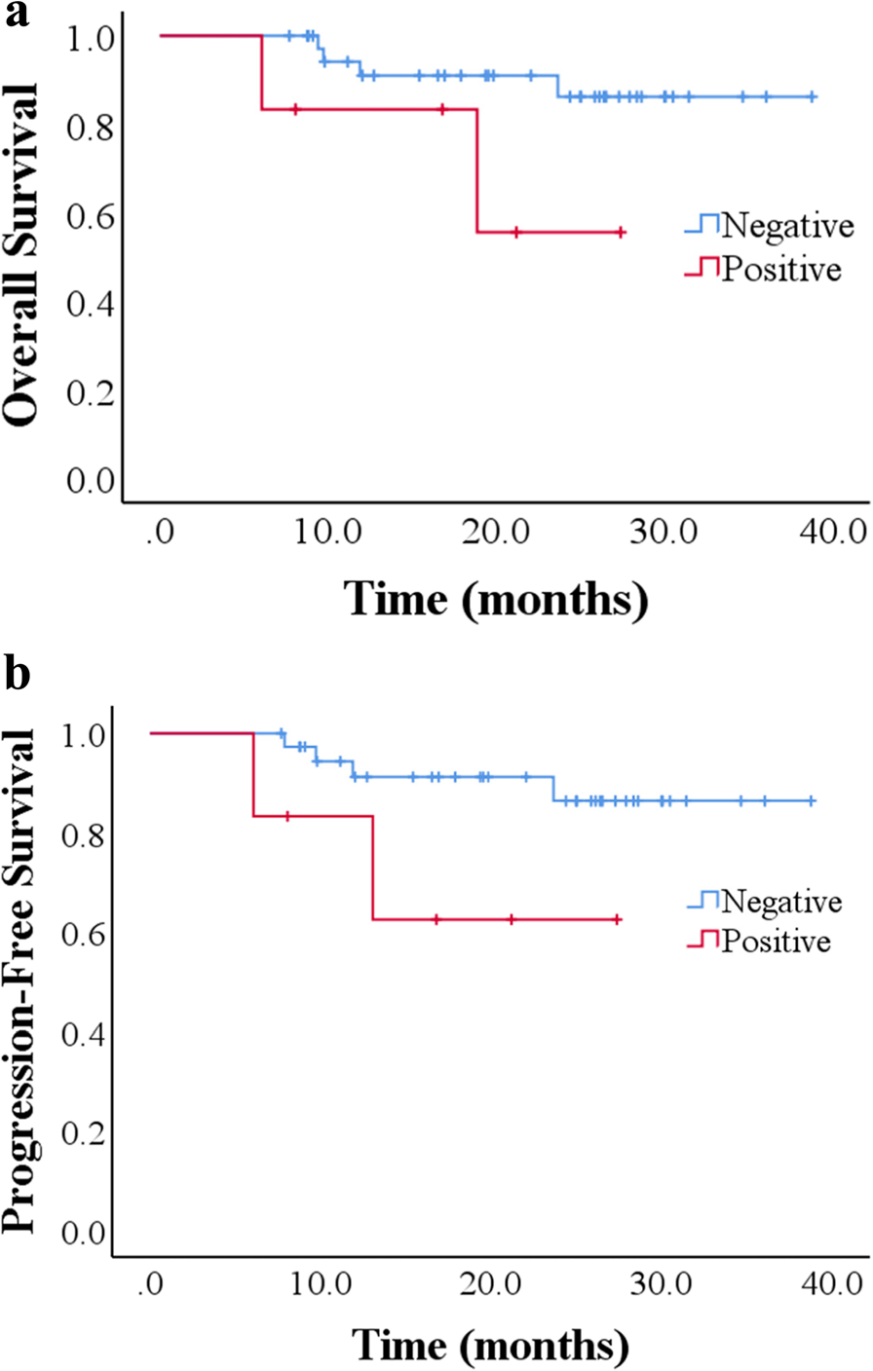
**a** Kaplan-Meier overall survival curve (*p* = 0.068); **b** Kaplan-Meier progression-free survival (*p* = 0.079). Patients were stratified by lymphatic/vascular invasion

On univariate analysis, pathologic regional lymph node status was not predictive of OS (*p* = 0.38). The 3-year estimations of OS were 85.6% and 88.9% for N0 and N1, respectively. The 2-year OS of 62.5% was estimated for the N2. The PFS estimations for the above were 91.7% and 88.9% for 3-year and 66.7% for 2-year (*p* = 0.373) (Fig. 2a, b). Lymphatic/vascular invasion also did not predict for OS (*p* = 0.068) or PFS (*p* = 0.079) in our study. The 3-year estimation of OS and PFS were 86.2% and 86.4% for lymphatic/vascular invasion negative. The 55.6% and 62.5% of 2-year OS and PFS were respectively estimated for the lymphatic/vascular invasion positive (Fig. 3a, b). The margins of our patients were negative. Therefore, no additional statistical analysis was performed.

We have performed statistical analysis on the influence of several disease factors and treatment parameters to the PFS and OS. The factors include age, gender, tumor size, number of positive lymph nodes, total number of lymph nodes examined, N stage (all patients are T3M0, only N stage is different), perineural invasion, lymphatic/vascular invasion, adjuvant chemotherapy, applicator size, IORT does, and time of IORT (Supplemental Table 1). None of the *r* values are higher than 0.3 or lower than − 0.3. Our data showed that none of any of other factors has association with the OS or PFS.

Perioperative 30-day mortality was 0%, while 7 complications were occurring in 5 patients. Early complications (≤ 30 days) occurred in 9% (*n* = 4) of patients, including wound infection 5% (*n* = 2), anastomotic fistula 2% (*n* = 1), and healing delay 2% (*n* = 1). Three late complications (> 30 days) occurred in 3 patients, giving a long-term morbidity rate of 7%. All 3 were related to small bowel obstruction. There was no severe toxicity (CTCAE grades 3 or 4) related to the multimodality therapy. Information on complications is described in Table 3.

**Table 3:** Early and late complications after the combination therapy of surgery and IORT

Complication	Total (number)
Early (30 days)	4
Mortality	0
Anastomotic leak/abscess/fistula	1
Small bowel obstruction	0
Wound infection or breakdown	2
Dehiscence	0
Ureteral injury	0
Others	1
Late(> 30 day)	3
Peripheral neuropathy	0
Small bowel obstruction	3
Ureteral obstruction	0
Wound infection/breakdown	0
Fistula with abscess	0
Bladder dysfunction	0
Sexual dysfunction	0
Enteritis/proctitis	0
Pelvic or abdominal abscess	0
There was no severe toxicity (CTCAE grades 3 or 4) related to the multimodality therapy	

## Discussion

Currently, there was very limited data available on IORT for colon cancer, especially for locally advanced colon cancer [20, 21]. A Russian literature showed their experience with 20 T3-4 colorectal adenocarcinoma patients using Intrabeam® PRS after curative surgery [17]. A dose of 14–17 Gy was distributed to the surface. Authors indicated the possibility of IORT to be used with curative resection with minimal early complications. For eradicating microscopic and subclinical disease after surgery, the surface dose ranged from 13 to 23 Gy according to a retrospective review of the Cleveland clinic experience for rectal cancer [18]. The study proved that Intrabeam® PRS, which provides IORT for patients with rectal cancer, seems to be a safe technique. This year, Sergey et al. reported that a single dose of 10–20 Gy for low-kV x-ray IORT was a valuable alternative for LACC patients in the absence of access to external beam radiotherapy (EBRT) [19]. Our IORT dose of 10–18 Gy was within the safe range based on the experience of other institutions. The final dose given is determined by the radiologist but requires, necessarily, a multidisciplinary collaboration with the surgeon and pathologist.

In our study, the present in-field local control was 100%. It was a very encouraging result. Additionally, based on current obtainable results, the 5-year local control was between 86 and 89% due to multimodality treatment including surgery, EBRT, and IOERT. Liska et al. found that the median time to LR was 21 months [5]. This was comparable to our median follow-up time. It was reasonable to infer that we achieved better locoregional control of 95%. Despite 19 of our patients received adjuvant chemotherapy according to standards, it was notable that adjuvant chemotherapy was not involved in reducing LR of patients with either stage II or stage III tumors [5]. The estimated 5-year OS was between 61 and 76%. We found that 3-year OS was 82.1%. Notably, at least two of the six patients did not die directly from colon cancer in our study. Therefore, the actual survival rate should be better than what we reported here in this study. Meanwhile, our 3-year PFS was 82.8%, better than the 43% in the early report [21]. The 5-year distant failure was 12% according to the previous data, which is much higher than our 5% [20].

In particular, extensive surgical resection is required for patients with LACC and this comes with a major risk of complications. Therefore, in the present era of increasing medical costs and outcome consciousness, it is essential to assess complications associated with the combination of low-kV x-ray IORT and surgery. Our results suggest that patients treated with low-kV x-ray IORT had encouraging PFS and OS and without an increase in short-term or long-term complications in comparison to previous multimodality studies, whose acute complications were not more than 10% and long-term morbidities were between 37 and 53% [22]. In our study, early complications occurred in 9% of patients, and 7% of patients had late complications, and surgery time was not extended significantly (mean low-kV x-ray IORT time = 15.7 min, range 9.2–26.6 min). Our analysis indicated that the addition of low-kV x-ray IORT to standard treatment led to better results with no increased toxicity.

As has been previously shown, postoperative regional lymph node status and lymphovascular invasion directly affected tumor stage and prognosis [23, 24]. The prognosis was very different from T3N0 to T3N2 patients. However, in our study, regional lymph node status and lymphovascular invasion had no significant impact on PFS or OS in patients with pT3 colon cancer. Although our analysis showed a trend, results did not get statistically significant differences. Our results suggested a potential role for low-kV x-ray IORT in the management of LACC, in particular, the setting of pT3 disease with pathologically involved lymph nodes and/or lymphovascular invasion positive patients. Our data showed that none of any of the other factors has association with the OS or PFS, confirming that OS and PFS in this study are the results from the treatment of low-kV x-ray, not from other sources. However, we cannot exclude the effect of limited follow-up time and the small patient number at present.

It was also notable that variations in the histology of our study included small intestinal neuroendocrine carcinoma, mucinous adenocarcinoma, and adenocarcinoma. Nevertheless, neuroendocrine tumors had a poor prognosis with 3-year survival was 15%, and five-year survival was 6%. Overall survival was poor especially for small-cell neuroendocrine carcinomas [25]. Comparing with non-mucinous adenocarcinoma, mucinous adenocarcinoma was a distinct subgroup of colon cancer with a worse prognosis [26]. Thus, instead of affecting our current results, it indicated that we achieved quite good results.

Our study has several limitations which include it being a retrospective, non-randomized, single-center study with no control group. There may also be a significant selection bias. The follow-up time is relatively insufficient. Because of current rare data on IORT for colon cancer, available results are relatively inadequate. This could limit the generalizability of results from this study to a larger population.

Despite these limitations, our results suggest a potential role for low-kV x-ray IORT in the management of LACC and achieve the effect of not being inferior to the electron IOERT without increasing toxicity. Larger prospective comparative analyses are needed to better evaluate outcomes for patients with LACC receiving low-kV x-ray IORT.

## Conclusion

Patients with LACC who have undergone an additional low-kV x-ray IORT can achieve encouraging locoregional control, PFS, OS, and distant control without an increase in short-term or long-term complications. Low-kV x-ray IORT can be considered as part of management in pT3 LACC. Further long-term follow-up is still needed.

## Supplementary information

**Additional file 1: Supplementary Table 1.** The correlation r values of OS and PFS with disease factors.

**Additional file 2.** Data collection.

## Data Availability

The datasets used and/or analyzed during the current study are available from the corresponding author on reasonable request.

## Abbreviations

LACC: Locally advanced colon cancer
kV: Kilovolt
IOERT: Electron beam intraoperative radiotherapy
OS: Overall survival
PFS: Progression-free survival
LR: Locoregional recurrence
IORT: Intraoperative radiotherapy
TNM: Tumor node metastasis
EBRT: External beam radiotherapy
