# Supplementary material for: Low-kilovolt x-ray intraoperative radiotherapy for pT3 locally advanced colon cancer: a single-institution retrospective analysis
Source: World J Surg Oncol. 2020 Jun 17;18:132. doi: 10.1186/s12957-020-01903-x (PMC7301558; doi:10.1186/s12957-020-01903-x)
Supplement: Supplementary file 1 — Additional file 1: Supplementary Table 1. The correlation r values of OS and PFS with disease factors. [file 12957_2020_1903_MOESM1_ESM.docx]

**Supplementary Table 1.** The correlation r values of OS and PFS with disease factors

|  | PFS | OS |
| --- | --- | --- |
| Age | -0.1 | -0.2 |
| Gender | 0 | 0 |
| Tumor size | -0.1 | -0.1 |
| Number of positive lymph nodes | -0.3 | -0.3 |
| Total number of lymph nodes examined | -0.3 | -0.3 |
| N stage | -0.1 | -0.1 |
| Perineural invasion | -0.2 | -0.2 |
| Lymphatic/vascular invasion | -0.2 | -0.2 |
| Adjuvant chemotherapy | 0.1 | 0.1 |
| Applicators size | 0.2 | 0.2 |
| IORT does | 0 | 0 |
| IORT time | 0 | 0 |
